# AI DEVELOPMENT AND AGING POPULATIONS: CRITICAL JUNCTURE OR PATH DEPENDENCY? POLICY ANALYSIS

**DOI:** 10.1093/geroni/igae098.1114

**Published:** 2024-12-31

**Authors:** Justyna Stypinska, Anrea Rosales, Maria Sourbati, Rüya Koçer, Halina Nadobnik

**Affiliations:** WZB Berlin Social Science Center, Berlin, Berlin, Germany; Open University of Catalonia, Barcelona, Catalonia, Spain; University of Brighton, Brighton, England, United Kingdom; Leiden University, Leiden, Friesland, Netherlands; WZB, Berlin, Berlin, Germany

## Abstract

This paper presents policy landscapes of five European countries - Germany, Holland, Spain, Poland, and Great Britain, as well as international documents (EU, UNESCO, Council of Europe) - scrutinizing their approaches to AI development through the lens of aging population. The research question addressed is: How are older persons represented and situated within AI governance in these national and international documents? The study, conducted as part of the “Ageism in AI (AGEAI)” research project, employs a policy text analysis to examine the explicit and implicit ways ageism manifests in AI development strategies. The research scrutinizes policy texts to discern the extent to which the needs, rights, wishes and perspectives of older individuals are acknowledged and integrated. The results show that economic growth, expected due to advancement of AI technologies, leaves a large segment of population – those not active in the labor market - beyond the scope of the policies. Furthermore, we observe diverse approaches and levels of inclusivity of the aging population across the five countries. While some nations exhibit explicit efforts to incorporate considerations for older adults within their AI policies, others demonstrate a significant gap in addressing age-related concerns. Moreover, the presentation will reflect on whether the direction of AI development proclaimed by the strategic documents reflects a pattern of path dependency of those policy instruments or suggests a more radical approach – a (new) critical juncture in development of technology for, by and with older adults.

